# Unravelling homeostasis effects of phosphorus and zinc nutrition by leaf photochemistry and metabolic adjustment in cotton plants

**DOI:** 10.1038/s41598-021-93396-1

**Published:** 2021-07-02

**Authors:** Elcio Ferreira Santos, Paula Pongrac, André Rodrigues Reis, Flávio Henrique Silveira Rabêlo, Ricardo Antunes Azevedo, Philip J. White, José Lavres

**Affiliations:** 1 Center for Nuclear Energy in Agriculture, University of São Paulo, Piracicaba, 13416-000 Brazil; 2 Biotechnical Faculty, University of Ljubljana, Jamnikarjeva 111, 1000 Ljubljana, Slovenia; 3 Jožef Stefan Institute, Jamova 39, 1000 Ljubljana, Slovenia; 4 São Paulo State University, Tupã, 17602-496 Brazil; 5 College of Agriculture Luiz de Queiroz, University of São Paulo, Piracicaba, 13418-900 Brazil; 6 Ecological Science Group, The James Hutton Institute, Invergowrie, Dundee, DD2 5DA UK; 7 Distinguished Scientist Fellowship Program, King Saud University, Riyadh, 11451 Saudi Arabia; 8 College of Resources and Environment, Huazhong Agricultural University, Wuhan, 430070 China

**Keywords:** Plant sciences, Plant physiology, Plant stress responses

## Abstract

Phosphorus (P) and zinc (Zn) uptake and its physiological use in plants are interconnected and are tightly controlled. However, there is still conflicting information about the interactions of these two nutrients, thus a better understanding of nutritional homeostasis is needed. The objective of this work was to evaluate responses of photosynthesis parameters, P-Zn nutritional homeostasis and antioxidant metabolism to variation in the P × Zn supply of cotton (*Gossypium hirsutum* L.). Plants were grown in pots and watered with nutrient solution containing combinations of P and Zn supply. An excess of either P or Zn limited plant growth, reduced photosynthesis-related parameters, and antioxidant scavenging enzymes. Phosphorus uptake favoured photochemical dissipation of energy decreasing oxidative stress, notably on Zn-well-nourished plants. On the other hand, excessive P uptake reduces Zn-shoot concentration and decreasing carbonic anhydrase activity. Adequate Zn supply facilitated adaptation responses to P deficiency, upregulating acid phosphatase activity, whereas Zn and P excess were alleviated by increasing P and Zn supply, respectively. Collectively, the results showed that inter ionic effects of P and Zn uptake affected light use and CO_2_ assimilation rate on photosynthesis, activation of antioxidant metabolism, acid phosphatase and carbonic anhydrase activities, and plant growth-related responses to different extents.

## Introduction

Phosphorus (P) and zinc (Zn) are essential elements for plants^1,2^. However, in most agricultural soils, the available concentrations of these nutrients are low, which causes Zn and/or P deficiencies in plants and reduces agricultural productivity^3^. However, since these mineral fertilisers are essentially non-renewable resources^4^, it is essential to optimise their use by plants, especially plants requiring large amounts of P and Zn, such as cotton (*Gossypium hirsutum* L.)^5,6^. Limitations to cotton cultivation due to inadequate supply of P and Zn are common^7^, especially in regions where there has been an intensification and increased productivity of cotton crops^6,8^.

The uptake and physiological utilisation of P and Zn by plants are regulated by a complex network of interrelated molecular, biochemical and physiological processes. The deficiency or excess of either of these nutrients can alter the uptake and physiological utilisation of the other, often in a species-specific manner^6,9–11^. In addition, the effect of P × Zn interactions can vary even between genotypes of the same species, as reported for *Brassica oleracea* L.^9,12^ and lettuce^13^. Seemingly contradictory information on P × Zn interactions limits our understanding of nutritional physiology, which can result in errors in crop management (e.g. fertiliser applications). The negative effects of Zn deficiency on P uptake, which result in the toxic accumulation of P in leaves, have been observed, for example, in maize (*Zea mays* L.)^14^, barley (*Hordeum vulgare* L.)^15^, lettuce (*Lactuca sativa* L.)^13^ and *Arabidopsis thaliana* L*.*^16^. These observations demonstrate that Zn-deficient plants lose the ability to regulate P uptake. Similarly, there are reports of P-deficient plants overaccumulating Zn^17–19^. Conversely, the excessive application of P tends to reduce the concentration of Zn in plants^9,11,20^. Responses to P × Zn interactions common to all plants are adjustments in antioxidant metabolism and photosynthetic processes^6^.

Zn deficiency inhibits the activities of a series of antioxidant enzymes, resulting in extensive oxidative damage to membrane lipids, proteins, chlorophyll and nucleic acids^20^. In addition, Zn deficiency reduces carbonic anhydrase activity, impairing the transfer of CO_2_/HCO_3_ in the leaf mesophyll for photosynthetic CO_2_ fixation^21,22^. Phosphorus-deficient plants, show reduced control of photosynthetic process and impaired energy metabolism, causing oxidative damage^23,24^. Zinc toxicity causes oxidative damage and impairs the photochemical and gas exchange relationships in plants^25,26^ Nevertheless, the physiological response of the P × Zn interaction in plants remains poorly understood. The study of integrative effects of P and Zn linking antioxidant metabolism, acid phosphatase and carbonic anhydrase activities and photosynthesis, help to clarify cross-talk between metabolic pathways of P and Zn.

In cotton plants an optimal P × Zn supply (4 mM P × 4 μM Zn) enabled greatest biomass accumulation, while an imbalanced supply of these nutrients led to Zn deficiency, P toxicity or Zn toxicity^6^. Furthermore, net photosynthetic rate, stomatal conductance, transpiration rate and instantaneous carboxylation efficiency increased as P or Zn supply increased. Building on this initial evaluation of the responses to a combination of P and Zn supply in cotton^6^, the aim of this work was to evaluate responses of photosynthesis-related parameters, P-Zn nutritional homeostasis, antioxidant protective metabolism and growth to variation in the P × Zn nutrition of cotton (*Gossypium hirsutum* L.).

## Results

### Phosphorus and Zn ion homeostasis

**Figure 1:**
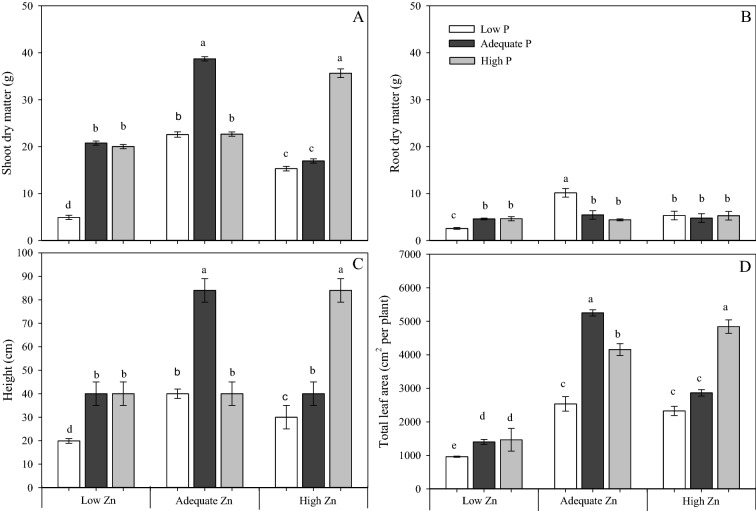
Shoot dry matter (**A**), root dry matter (**B**), plant height (**C**) and total leaf area (**D**) of cotton (*Gossypium hirsutum*) plants grown in nutrient solution containing different concentrations of phosphorus (P) and zinc (Zn) for 90 days. Low P = 0.5 mM; Adequate P = 4 mM; High P = 8 mM; Low Zn = 0.5 µM; Adequate Zn = 4 µM; High Zn = 8 µM. Different letters indicate significant differences for each dependent variable separately (Tukey test at *P* ≤ 0.05; n = 4). Sigma Plot 11 software (SYSTAT, San Jose, CA, USA) was used to display data.

All growth parameters measured (shoot and root biomass, plant height and total leaf area) were smallest when cotton plants received the Low P × Low Zn supply (0.5 mM P × 0.5 µM Zn; Fig. 1). When the P supply was low, these parameters increased significantly upon increasing Zn supply. Root biomass exhibited the smallest response to the P × Zn treatments (Fig. 1B). By contrast, the shoot biomass, the plant height and the total leaf area (Fig. 1A,C,D) were greater when plants received an Adequate or High P supply (4 mM and 8 mM, respectively) than when they received a Low P supply. At the High Zn supply (8 µM) the aboveground traits were greater when plants received a High P supply compared to a Low or Adequate P supply.

**Figure 2:**
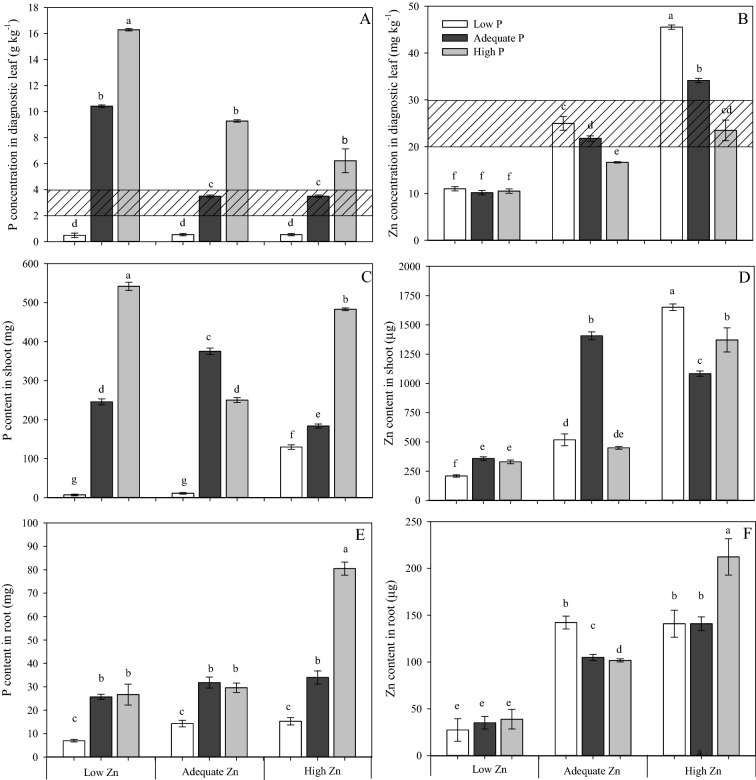
Phosphorus (P) concentration in diagnostic leaf (fifth leaf from the top) (**A**), zinc (Zn) concentration (**B**), P content in shoot (**C**), Zn content in shoot (**D**), P content in root (**E**) and Zn content in root (**F**) of cotton (*Gossypium hirsutum*) plants grown in nutrient solution containing different concentrations P and Zn for 90 days. Low P = 0.5 mM; Adequate P = 4 mM; High P = 8 mM; Low Zn = 0.5 µM; Adequate Zn = 4 µM; High Zn = 8 µM. Different letters indicate significant differences for each dependent variable separately (Tukey test at *P* ≤ 0.05; n = 4). (Filled Square) Sufficiency rage for cotton suggested by Serra et al. (2012). Sigma Plot 11 software (SYSTAT, San Jose, CA, USA) was used to display data.

The concentration of P in the diagnostic leaf increased with increasing P supply (Fig. 2A). The largest concentration of P in the diagnostic leaf was measured in the High P × Low Zn treatment (i.e. 8 mM × 0.5 µM). Plants grown with Low P (0.5 mM) had the smallest P concentration irrespective of the Zn supply. This P concentration was below the range of P sufficiency suggested by Serra et al^27^ for cotton, which implies that these plants were P deficient. In the Adequate P and High P treatments (4 and 8 mM, respectively), increasing Zn supply reduced the P concentration in diagnostic leaves.

The concentration of Zn in the diagnostic leaf increased with increasing Zn supply (Fig. 2B), with the smallest Zn concentrations being measured in plants receiving Low Zn (0.5 µM). These Zn concentrations were below the range of Zn sufficiency suggested by Serra et al. (2012) for cotton, which implies that these were Zn-deficient plants. In plants grown in Adequate and High Zn treatments (4 and 8 µM, respectively) Zn concentrations in diagnostic leaves decreased with increasing P supply, but in the Low Zn treatment the P supply did not influence the Zn concentration of diagnostic leaves.

The P content of shoots (Fig. 2C) generally paralleled the P concentrations in diagnostic leaves (Fig. 2A), except in plants grown with Adequate P × Adequate Zn supply (4 mM P × 4 µM Zn). Comparing the plants receiving Adequate Zn supply (4 µM), the P content was greater in plants receiving Adequate P supply (4 mM) than plants receiving High P supply (8 mM). The largest P content was found in plants receiving High P × Low Zn supply (8 mM P × 0.5 µM Zn).

The Zn content of shoots was largest in plants cultivated with Low P × High Zn (0.5 mM P × 8 µM Zn) (Fig. 2D). Plants grown with Adequate P × Low Zn (4 mM P × 0.5 µM Zn) had smaller Zn concentrations in diagnostic leaves (Fig. 2B) and accumulated less Zn in shoots (Fig. 2D) than plants receiving High P × Adequate Zn supply (8 mM × 4 µM).

The P content of roots was smallest in plants grown with Low P (0.5 mM), regardless of the concentration of Zn in the solution (Fig. 2E). Plants grown with High P × High Zn (8 mM P × 8 µM Zn) had the largest root P content. Similarly, the largest accumulation of Zn in the roots (62% greater than that of plants grown in Adequate Zn supply) was found in plants receiving High P × High Zn supply (8 mM P × 8 µM Zn). The smallest root Zn content was found in plants receiving a Low Zn supply (0.5 µM), regardless of the P supply.

There was no effect of P or Zn supply on the concentrations of macronutrients and micronutrients in diagnostic leaves (Table S1), shoot and roots (Table S2), although P × Zn supply affected the concentrations of P and Zn in shoots and roots in a manner that paralleled the P contents of shoots and roots (Fig. 2).

### Physiology responses

**Figure 3:**
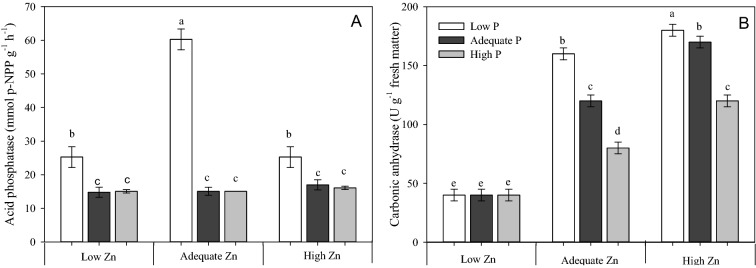
Acid phosphatase activity (**A**) and carbonic anhydrase activity (**B**) in leaves of cotton (*Gossypium hirsutum*) plants grown in nutrient solution containing different concentrations phosphorus (P) and zinc (Zn) for 90 days. Low P = 0.5 mM; Adequate P = 4 mM; High P = 8 mM; Low Zn = 0.5 µM; Adequate Zn = 4 µM; High Zn = 8 µM. Different letters indicate significant differences for each dependent variable separately (Tukey test at *P* ≤ 0.05; n = 4). Sigma Plot 11 software (SYSTAT, San Jose, CA, USA) was used to display data.

Acid phosphatase activity was largest in plants receiving Low P × Adequate Zn supply (0.5 mM P × 4 µM Zn; Fig. 3A). There was threefold more acid phosphatase activity in plants grown with Low P × Adequate Zn compared to plants grown with Low P × Low Zn (i.e. 0.5 mM P × 2 µM Zn) or Low P × High Zn (i.e. 0.5 mM P × 8 µM Zn). The activity of carbonic anhydrase (Fig. 3B) paralleled the Zn concentration of diagnostic leaves (Fig. 2B). The activity of carbonic anhydrase was smallest in plants grown with Low Zn (0.5 µM), regardless of the P supply, and in plants grown in Adequate and High Zn treatments (4 and 8 µM, respectively) carbonic anhydrase activity decreased with increasing P supply.

**Figure 4:**
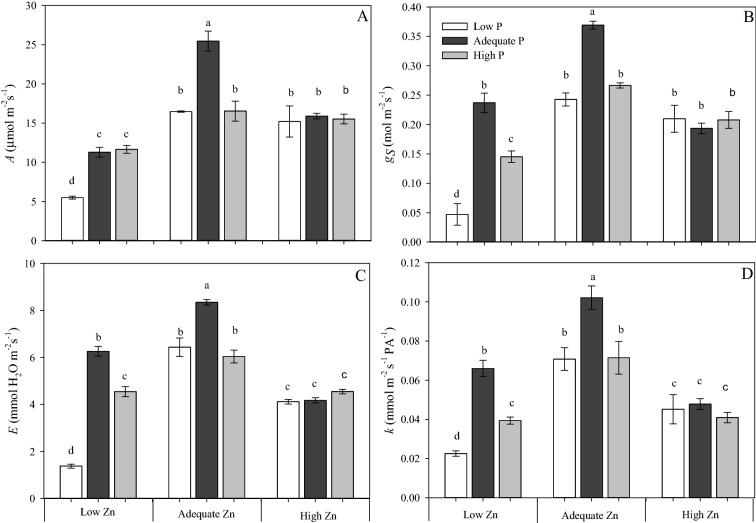
Net photosynthesis rate (*A*) (**A**), stomatal conductance (*gs*) (**B**), transpiration (*E*) (**C**) and instantaneous carboxylation efficiency (*k* = *A/Ci*) (**D**) measured on the diagnostic leaf (fifth leaf from the top) of cotton (*Gossypium hirsutum*) plants grown in nutrient solution containing different concentrations of phosphorus (P) and zinc (Zn) for 90 days. Low P = 0.5 mM; Adequate P = 4 mM; High P = 8 mM; Low Zn = 0.5 µM; Adequate Zn = 4 µM; High Zn = 8 µM. Different letters indicate significant differences for each dependent variable separately (Tukey test at *P* ≤ 0.05; n = 4). Sigma Plot 11 software (SYSTAT, San Jose, CA, USA) was used to display data.

The largest values for *A*, *g*_*S*_, *E* and *k* (Fig. 4) were observed in plants receiving Adequate P × Adequate Zn supply (4 mM P × 4 µM Zn). The smallest values for all these traits were observed in plants grown with Low P × Low Zn supply (0.5 mM P × 0.5 µM Zn). The P supply had no effect on gas exchange or photochemistry in the High Zn (8 µM) treatments.

**Figure 5:**
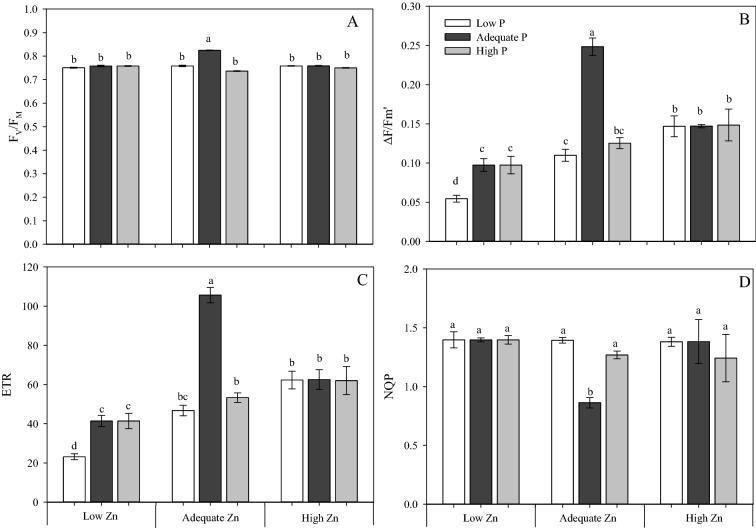
Maximum quantum efficiency of photosystem II (Fv/Fm) (**A**), effective quantum efficiency of photosystem II (∆F/F mʹ) (**B**), apparent electron transport rate (ETR) (**C**), non-photochemical fluorescence quenching *(NQP)* (**D**) measured on the diagnostic leaf (fifth leaf from the top) of cotton (*Gossypium hirsutum*) plants grown in nutrient solution containing different concentrations of phosphorus (P) and zinc (Zn) for 90 days. Low P = 0.5 mM; Adequate P = 4 mM; High P = 8 mM; Low Zn = 0.5 µM; Adequate Zn = 4 µM; High Zn = 8 µM. Different letters indicate significant differences for each dependent variable separately (Tukey test at *P* ≤ 0.05; n = 4). Sigma Plot 11 software (SYSTAT, San Jose, CA, USA) was used to display data.

Plants receiving Adequate P × Adequate Zn supply (4 mM P × 4 µM Zn) had maximum quantum efficiency of photosystem II—[Fv/Fm] (Fig. 5A); effective quantum efficiency of photosystem II—[∆F/Fm’] (Fig. 5B) and the apparent electron transport rate through photosystem II − [ETR] (Fig. 5C), but the smallest non-photochemical extinction coefficient – [NPQ] (Fig. 5D) compared to all other treatments.

**Figure 6:**
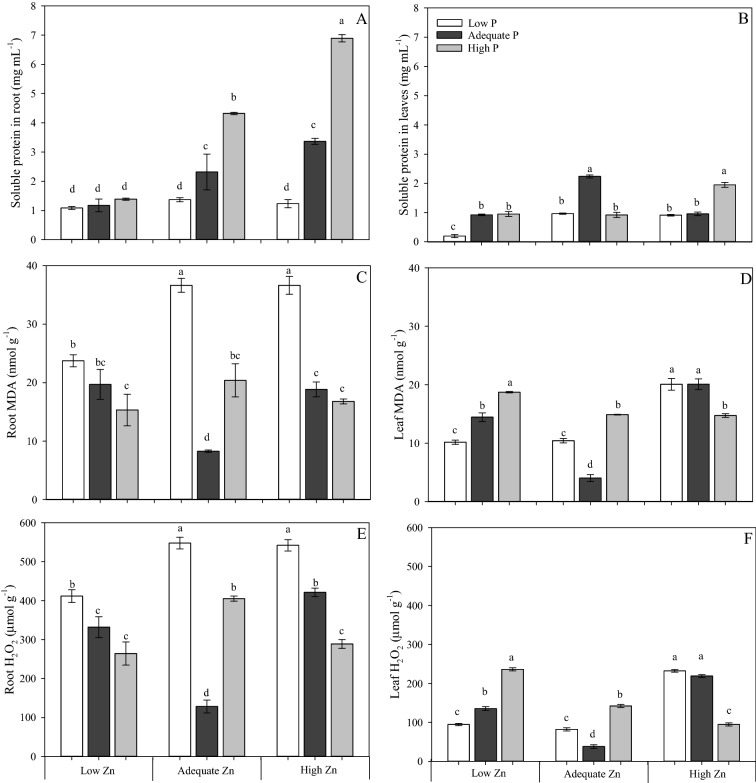
Concentration of soluble protein in roots (**A**) and in leaves (**B**), concentration of malondialdehyde (MDA) in roots (**C**) and in leaves (**D**), and concentration of hydrogen peroxide (H_2_O_2_) in roots (**E**) and in leaves (**F**) of cotton (*Gossypium hirsutum*) plants grown in nutrient solution containing different concentrations of phosphorus (P) and zinc (Zn) for 90 days. Low P = 0.5 mM; Adequate P = 4 mM; High P = 8 mM; Low Zn = 0.5 µM; Adequate Zn = 4 µM; High Zn = 8 µM. Different letters indicate significant differences for each dependent variable separately (Tukey test at *P* ≤ 0.05; n = 4). Sigma Plot 11 software (SYSTAT, San Jose, CA, USA) was used to display data.

In general, roots had larger concentrations of soluble proteins, exhibited stronger root peroxidation and had larger H_2_O_2_ concentration than shoots (Fig. 6). Nevertheless, the responses of roots paralleled those of leaves, except in the concentration of soluble proteins (Fig. 6A,C,E). The largest concentration of total soluble proteins in the leaves was observed in plants grown with Adequate P × Adequate Zn supply (4 mM P × 4 µM Zn) and was accompanied by the weakest lipid peroxidation and the smallest H_2_O_2_ concentration (Fig. 6B,D,F). The concentration of soluble proteins in roots was increased by increasing P supply, buy only in plants receiving the Adequate and High Zn supply (4 µM and 8 µM, respectively; Fig. 6B).

**Figure 7:**
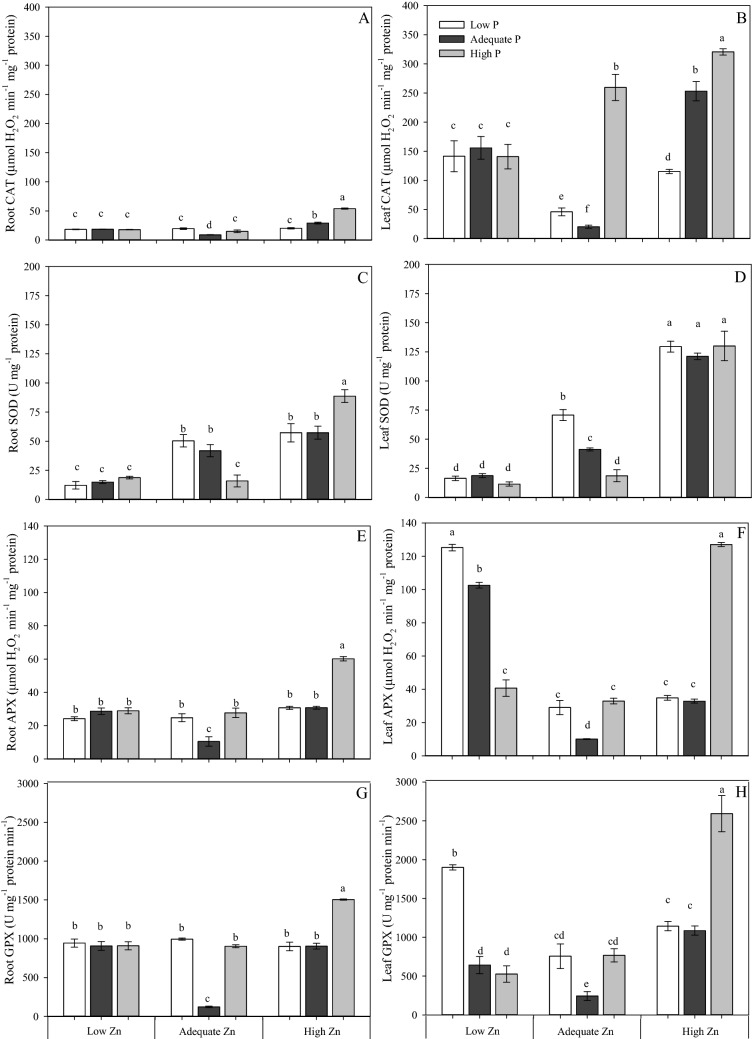
Catalase activity (CAT) in roots (**A**) and in leaves (**B**), superoxide dismutase activity (SOD) in roots (**C**) and in leaves (**D**), ascorbate peroxidase activity (APX) in roots (**E**) and in leaves (**F**), and guaiacol peroxidase activity (GPX) in roots (**G**) and in leaves (**H**) of cotton (*Gossypium hirsutum*) plants grown in nutrient solution containing different concentrations of phosphorus (P) and zinc (Zn) for 90 days. Low P = 0.5 mM; Adequate P = 4 mM; High P = 8 mM; Low Zn = 0.5 µM; Adequate Zn = 4 µM; High Zn = 8 µM. Different letters indicate significant differences for each dependent variable separately (Tukey test at *P* ≤ 0.05; n = 4). Sigma Plot 11 software (SYSTAT, San Jose, CA, USA) was used to display data.

There was no significant effect of P supply on the activity of the four antioxidative enzymes studied (CAT, SOD, APX, GPX) in roots when plants were grown with Low Zn (0.5 µM), but when plants received a High Zn supply (8 µM), there was an increase in activity with increasing P supply (Fig. 7A,C,E,G). In shoots, increasing P supply reduced the activities of APX and GPX in plants receiving Low Zn (0.5 µM) supply, while activities of CAT and SOD were not affected by P supply in the Low Zn treatment (Fig. 7B,D,F,H). Except for SOD, activities of antioxidant enzymes in leaves were increased by increasing P supply in plants receiving High Zn (8 µM). For CAT, APX and GPX the smallest activity was measured in the Adequate P × Adequate Zn (4 mM P × 4 µM Zn) in both leaves and roots.

### Pearson correlation

**Figure 8:**
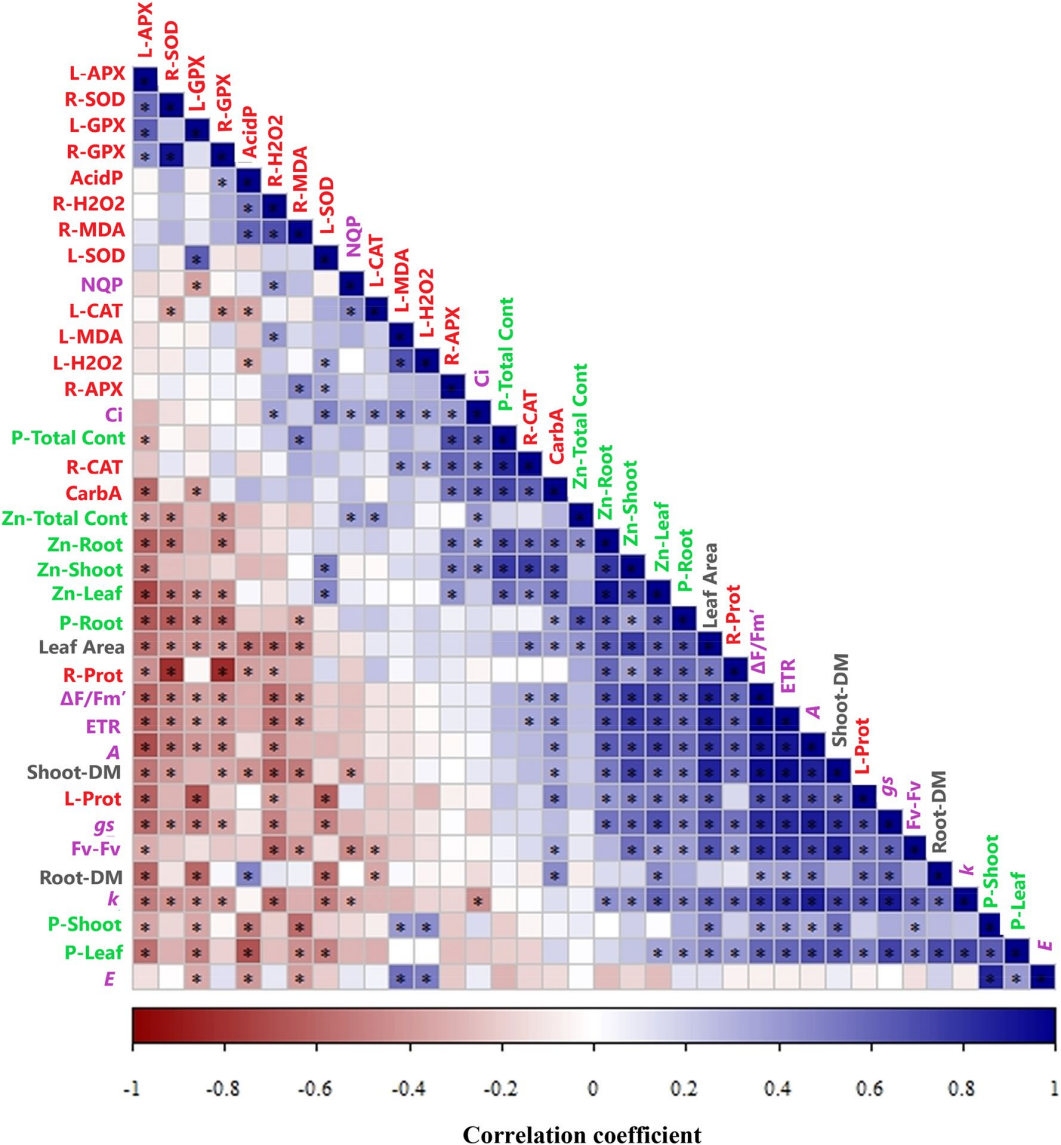
Heatmap of Pearson correlation coefficients between variables measured in cotton (*Gossypium hirsutum*) plants grown in nutrient solution containing different concentrations of phosphorus (P) and zinc (Zn) for 13 weeks. *Indicates significant correlation (at *P* ≤ 0.05). Traits associated with P and Zn concentration and content are highlighted in green, parameters related to growth and biomass are highlighted in grey, gas exchange and photochemistry parameters are highlighted in purple and enzyme activities are highlighted in red. Figures were produced using R (v3.4.3, https://www.R-project.org).

Shoot and root dry mass correlated positively with P and Zn concentration in diagnostic leaf (Fig. 8). In addition, P and Zn concentration and content were positively correlated with leaf gas exchange and photochemical parameters. These results highlight the importance of P and Zn nutrition for photosynthetic performance and biomass accumulation. As expected, there was a negative correlation between acid phosphatase activity and P concentration in leaves and a positive correlation between carbonic anhydrase activity and Zn concentration in leaves. Indicators of oxidative stress were negatively correlated with P and Zn concentration and content, suggesting that P and Zn deficiencies trigger an increase in the activity of antioxidant enzymes.

## Discussion

Adaptation responses of plants to nutrient deficiencies are constantly being fine-tuned to ensure minimal needs are met whilst satisfying carbon cost trade-offs^6^. Judging from leaf P and Zn concentrations in the experiment reported here, minimal needs for P were not met in cotton plants receiving the Low P supply and minimal needs for Zn were not met in the Low Zn treatment. Phosphorus deficiency in plants is very common problem that restricts the growth and productivity of crops around the world^28,29^. Under severe P deficiency Rubisco activity is limited^23,24^ and leaf ultrastructure is affected^30^. It is likely that the resulting oxidative stress in leaves was mitigated mainly by two antioxidative enzymes, APX and GPX, whose activities were larger than other enzymes and correlated negatively with shoot biomass and concentrations of P and Zn in leaves, as well as the photosynthesis parameters (Fig. 8). Increased activities of antioxidant enzymes are common in plants in response to the ROS (reactive oxygen species) generated by P deficiency^23^. The increased activities of APX and GPX presumably tried to quench the production of H_2_O_2_ in the leaves of plants grown in the Low P supply.

Phosphorus deficiency in plants reduce the ability to process light energy, which may cause production of reactive oxygen species in chloroplasts and the consequent activation of antioxidant system in chloroplasts which is scavenged by the sequential electron transfer from ascorbate catalysed by APX and GPX^23^. A significant negative correlation was observed between the concentration of P in the leaf and APX and GPX activities (Fig. 8). In this way, the responses of the antioxidant system are explained by the concentration of P or Zn in cotton leaves and corresponding changes in photosynthesis. Similarly, Low Zn supply increased the APX and GPX activities in cotton leaves. Zn deficiency also causes the production of ROS in chloroplasts and decreased activity of SOD (zinc-dependent enzyme) and the consequent activation of antioxidant mechanisms in chloroplasts by APX and GPX^26^.

The results presented here suggest that the oxidative stress resulting from P toxicity in leaves was mitigated (upregulated) mainly by CAT in the High P × Adequate Zn treatment; and by APX, GPX and SOD in the High P × High Zn treatment (Fig. 6). CAT is frequently used by cells to rapidly catalyse the decomposition of ROS into gaseous oxygen and water^31^. The results showed that Low P supply, accompanied by adequate Zn in concentration in cotton leaves, resulting in a rapid response of the antioxidative system by CAT. On the other hand, High P × High Zn treatment stimulate a coordinated increase in enzymatic antioxidants (SOD, APX and GPX) to attenuate oxidative stress. Probably, highest concentrations of Zn in the leaves stimulate this response of the antioxidant system, as already observed to *Solanum lycopersicum* exposed to high concentrations of Zn in the cultivation^25^. Different responses of the antioxidative system were influenced by P and Zn concentration in the leaves imposed by the treatments. Intensive research of P × Zn interaction on antioxidant systems, helps us understand the different mechanisms involved in cell stress responses and increase more insight into the antioxidative responses induced by P and Zn stress.

Plants grown in Low P x Adequate Zn treatment, had greater acid phosphatase activity, which may be an adaptation response to P deficiency by remobilising P from cellular sources^2,28^. Interestingly, the largest root biomass was observed in plants grown in the Low P × Adequate Zn treatment, suggesting that an adaptation increase in root biomass occurs as a response to P deficiency, but only when Zn supply is adequate. These observations are consistent with previous reports that P deficiency stimulates root growth^32,33^, which enables root foraging for P in soil, but only at adequate Zn supply^34^.

Historically, research has focused on optimizing P use efficiency independently^2,35^, or in association with nitrogen and potassium inputs^36^. The interactions of P with other nutrients have been generally neglected in agricultural management strategies. The results presented here indicate that an appropriate Zn supply is crucial for obtaining maximal P utilisation and yield. Thus, strategies that focus on balanced nutrient management could reduce P-fertiliser inputs and mitigate environmental damage and future P scarcity.

The extent of Zn uptake by cotton plants was affected primarily by Zn supply. Cotton plants receiving a Low Zn supply had the smallest Zn concentrations in diagnostic leaves and impaired photochemical parameters, indicating Zn deficiency. Zinc deficiency has been observed to reduce chlorophyll biosynthesis and Rubisco carboxylation capacity and alter stomatal ultrastructure^37^. Zinc deficiency also reduces carbonic anhydrase activity^21,22,38–40^. Thus, it is likely that the low leaf gas exchange and small carbonic anhydrase activity in the Low Zn treatment was a consequence of Zn deficiency. Carbonic anhydrase activity was strongly and positively correlated with Zn concentration and content in shoot (Fig. 8).

Zinc concentration was reduced by increasing P supply in plants grown with Adequate or High Zn supply, which indicates strong Zn × P interactions. This is in agreement with reports that increasing P supply reduced shoot Zn concentration in several crops^9,10,41^. However, a reduction in shoot Zn concentration in response to increasing P supply was not observed in experiments performed in nutrient solutions^42^, including those with cotton^20^. Ova et al.^10^ observed that the reduction in Zn uptake by wheat as P supply was increased only in soil-pot experiment and suggested that it was associated with a reduction in the colonization of roots by mycorrhizal fungi as a consequence of improved plant P nutrition^43^.

Phosphorus concentration in leaves of plants grown in the Low Zn × High P treatment exceeded the P toxicity threshold suggested by Serra et al.^27^ for cotton (4 g P kg^−1^ DW), indicating that Zn starvation results in P overaccumulation in cotton, which can induce P toxicity symptoms at high P supply. Excessive P in plants is uncommon, but some species, especially those in the Proteaceae family, have poor capacity to regulate P uptake, exhibiting symptoms of P toxicity in natural environments^44,45^. Zinc plays a specific role in the signal transduction pathway responsible for regulating genes associated with P uptake^15,16,34^ and plants lacking sufficient Zn lose the ability to regulate P uptake^13,34,46^.

In cotton plants, P toxicity was accompanied by reduced gas exchange and impaired photochemical reactions. Similar observations have been made in other plant species and it is possible that this effect is mediated by regulating the expression of genes encoding P transporters by plant Zn-nutritional status^9,34,46,47^. Consistent with this hypothesis, Huang et al.^15^ reported that Zn deficiency resulted in increased expression of genes encoding high affinity P uptake transporters in roots, irrespective of P supply. In addition, Zn deficiency also increases the expression of transporters loading P into the xylem, intensifying the P transport to the shoot^16^.

Plants grown with the High Zn × Low P treatment had leaf Zn concentrations exceeding the Zn concentration threshold suggested by Serra et al.^27^ for toxicity in cotton (30 mg Zn kg^−1^ DW). Similarly, plants grown with a High P supply had leaf P concentrations exceeding the P concentration threshold suggested by Serra et al.^27^ for toxicity in cotton (4 g Zn kg^−1^ DW). However, plants receiving High P × High Zn had much lower leaf Zn and P concentrations and did not show any significant reduction of shoot biomass, indicating that Zn and P toxicities can be alleviated increasing P and Zn supply, respectively.

In this study, the largest P and Zn content in the roots was found in plants receiving the High P × High Zn treatment, suggesting that the root restricted the transport of excess P and Zn to the shoot in this treatment. Root cells are believed to actively remove, sequester or immobilise potentially toxic elements to prevent their movement to the shoot^31,48^. It is possible that when both P and Zn supply are high Zn-phosphates might be formed in the apoplast of the root, which reduce the transport of Zn and P between roots and shoots^20^.

## Conclusion

The results demonstrate strong interactions between P and Zn nutrition of cotton plants. The excess or scarcity of P and Zn limited the growth of cotton plants and the P and Zn concentration in diagnostic leaves correlated with the physiological responses. Zinc uptake was affected by P supply and P uptake was affected by Zn supply. In particular, Zn and P toxicities were alleviated by increasing P and Zn supply, respectively. This work presents important results for the management of P × Zn interactions in cotton for improved utilisation of P and Zn resources, plant growth and, ultimately, greater yields.

## Methods

### Plant material and experimental conditions

The study was carried out under greenhouse conditions. The air temperature ranged between 23.2 °C (minimum) and 32.4 °C (maximum), with an average of 30.1 °C. The average air relative humidity was 65% and the maximum photosynthetic photon flux density (sunlight) was approximately 1700 µmol m^−2^ s^−1^, with a photoperiod of 12 h. Seeds of cotton (*Gossypium hirsutum* L. cv. FMT 709) were obtained from a seed distributor in Piracicaba city, Brazil. Seeds of cotton were germinated in vermiculite in plastic pots. When seedlings were five cm tall they were transferred to a 40-L plastic container filled with ¼-strength Hoagland nutrient solution according to Santos et al.^6^.

After one week, seedlings of equal shoot length (~ 10 cm) were transferred to individual containers (3 L; one plant per container), where they were grown in full strength Hoagland nutrient solution containing 12.0 mM N-NO_3_^−^, 4.0 mM N-NH_4_^+^, 6.0 mM K, 4.0 mM Ca, 2.0 mM Mg, 2.0 mM S, 50.0 µM Cl, 53.7 µM Fe, 25.0 µM B, 2.0 µM Mn, 0.5 µM Cu, and 0.5 µM Mo and combinations of three P concentrations (supplied as KH_2_PO_4_): Low P (0.5 mM), Adequate P (4.0 mM—control) and High P (8.0 mM) and three Zn concentrations (supplied as ZnCl_2_): Low Zn (0.5 µM); Adequate Zn (4.0 µM—control) and High Zn (8.0 µM), in the nutrient solution. The treatments were selected based on the results of Santos et al.^6^. The experiment was set in a 3 × 3 factorial arrangement adopting a randomized complete block design with four replicates. The nutrient solutions were continuously aerated, and pH was kept at 6.0 ± 0.5 using NaOH (1 M) or HCl (1 M). Nutrient solutions were renewed every 7 days.

The experiment was terminated on the first day of blooming, on the 90th day of treatment. Just before harvest, gas exchange and chlorophyll fluorescence parameters were measured (described in detail below), and the height of plants was determined. At harvest, plants were separated into shoots (leaves and stems), roots (washed immediately in running tap water) and diagnostic leaves (fourth fully expanded leaf counting from the apex of the main stem)^8^. Surface areas of all newly expanded and mature leaves per plant were measured using a digital area meter (LICOR LI-3100, Lincoln, NE, USA). Leaves and root subsamples (1 g of fresh weight per plant) were frozen in liquid nitrogen and stored in a freezer at -80ºC for the evaluation of the enzyme activities, as described in detail below. The remaining shoots and roots were dried at 60 °C for 10 days and their dry weights were determined and stored for mineral element analysis.

### Leaf chlorophyll fluorescence and gas exchange parameters

Chlorophyll fluorescence and gas exchange parameters were performed with an infrared gas analyzer (LI-6400XT, LICOR, Lincoln NE, USA) coupled to a modulated fluorometer (6400–40 LCF, LICOR, Lincoln NE, USA) between 08:00 and 10:00 h at a photon flux density (PPFD) of 1700 μmolm^−2^ s^−1^ and an air CO_2_ concentration of 380 μmol mol^−1^^49,50^ on the same day that the plants were harvested. Fluorescence maximal (Fm), fluorescence minimal (F0) and fluorescence variable (Fv = Fm–F0) were determined in dark-adapted diagnostic leaves (30 min). Fluorescence steady-state (Fs) and fluorescence maximal (Fmʹ) were assessed in light-adapted tissues. Based in this results, some photochemical variables were estimated according to Baker^51^: non-photochemical extinction coefficient [NPQ = (Fm − Fmʹ)/Fmʹ]; effective quantum efficiency of photosystem II [∆F/Fmʹ = (Fmʹ − Fs)/Fmʹ]; apparent electron transport rate through photosystem II [ETR = PPFD × ∆F/Fmʹ × 0.5 × 0.84]; and maximum quantum efficiency of photosystem II [Fv/Fm]. Leaf stomatal conductance (*g*_*S*_); transpiration (*E*); CO_2_ assimilation (*A*); and intercellular CO_2_ concentration (Ci); and instantaneous carboxylation efficiency (k = *A*/Ci) were determined^52^.

### Acid phosphatase assay (EC. 3.1.3.2)

Phosphatase acid activity was determined as described by Raposo et al.^53^. Frozen samples of leaves (50 mg) were incubated in 8 mL of a mixture containing 250 µM p-nitrophenyl phosphate and 0.1 mol L^−1^ sodium acetate buffer (pH 4.0), and kept at 30 °C in a water bath for 30 min. Subsequently, the extract was centrifuged at 10,000 g for 5 min at 4 °C. An aliquot of 5 mL was taken from the supernatant and then 2 mL of 2 M sodium hydroxide (NaOH) was added, and absorbance was read using a spectrophotometer (Perkin Elmer Lambda 40 UV VIS, Norwalk, USA) at 490 nm. The enzyme activity was expressed as µmol of hydrolyzed p- NPP substrate per hour, per fresh weight (µmol h^−1^ g^−1^).

### Carbonic anhydrase assay (EC.4.2.1.1)

Carbonic anhydrase activity was determined as reported by Wilbur and Anderson^54^. Leaves frozen samples (50 mg) were homogenized in 20 mM Tris HCl buffer (pH 8.3) containing 1 mM ethylenediamine tetraacetic acid (EDTA) and 10 mM 2-mercaptoethanol and centrifuged at 15,000 g for 5 min at 4 °C. Enzyme activity was measured by adding 100 µL of the leaf extract to 6 mL of 20 mM Tris HCl buffer (pH 8.3). The reaction was initiated with the addition of 4 mL of CO_2_-saturated water and was recorded the time needed for pH to drop from 8.3 to 6.3 in the presence (TP) and absence (TA) of enzyme. The enzyme activity was calculated by Wilbur and Anderson^54^ [2(TA − TP)/TA (g Fresh Weight)] and expressed as Units g^−1^ FW.

### Concentration of malondialdehyde (MDA) and H_2_O_2_

Concentration of MDA was used as a measure of lipid peroxidation in leaves and roots. Frozen samples (0.2 g) were homogenized in 2 mL of 0.1% (w/v) trichloroacetic acid (TCA) in the presence of 20% (w/w) of polyvinyl polypyrrolidone and homogenized. The extract was centrifuged at 10,000 g for 5 min at 4 °C. Following centrifugation, the supernatant (0.25 mL) was added to 1 mL of 20% (w/v) TCA containing 0.5% thiobarbituric acid. The mixture was maintained in a water bath at 95 °C for 30 min and then on ice for 20 min. The concentration of MDA was determined by the equation reported by Alcântara et al.^55^. The absorbance was read in a spectrophotometer (Perkin Elmer Lambda 40 UV VIS, Norwalk, USA) at 535 nm and 600 nm. The initial procedures for measuring the concentration of H_2_O_2_ were the same as described above for MDA measurements. Following centrifugation, the concentration of H_2_O_2_ was measured by adding 0.2 mL of supernatant to 0.2 mL of 100 mM potassium phosphate buffer (pH 7.0) and 0.8 mL of 1 mM potassium iodide. The solution was kept for one h in the dark and the absorbance was read in a spectrophotometer at 390 nm as described by Alexieva et al.^56^.

### Protein extraction and activity of antioxidant enzymes

Protein extraction was performed as described by Silva et al.^57^. In short, fresh leaves and roots (each 0.2 g) were homogenized in a chilled mortar with a pestle using a chilled extraction buffer containing 100 mM potassium phosphate buffer (pH 7.5), 1 mM ethylenediamine tetraacetic acid (EDTA), 3 mM DL-dithiothreitol and 5% (w/v) insoluble polyvinylpolypyrrolidone in 3:1 and 2:1 volume/fresh weight ratio to leaves and roots, respectively. The homogenate was centrifuged at 10,000 g for 30 min at 4 °C, and the supernatant was frozen in liquid nitrogen and stored at − 80 ºC until analyses. The protein concentration was determined in the extract following the method of Bradford using bovine serum albumin as a standard^58^. Superoxide dismutase (SOD; EC 1.15.1.1) activity was determined spectrophotometrically as described by Giannopolitis and Ries^59^. Catalase (CAT, EC. 1.11.1.6) activity was determined by evaluating the degradation of H_2_O_2_ at 240 nm over 1 min as described in detail by Silva et al.^58^. Ascorbate peroxidase (APX, EC. 1.11.1.11) activity was determined by assessing the rate of ascorbate oxidation at 290 nm at 30 ºC using a spectrophotometer as described previously by Silva et al.^58^. Guaiacol peroxidase (GPX, EC.1.11.1.7) activity was measured as described by Matsuno and Uritani^60^.

### Mineral element analysis

Concentrations of nitrogen (N), P, potassium (K), calcium (Ca), magnesium (Mg), sulphur (S), boron (B), copper (Cu), iron (Fe), manganese (Mn), and Zn were determined in diagnostic leaf, shoot and root samples. Nitrogen concentration was determined using the micro-Kjeldahl analytical method after sulphuric acid digestion of plant material as described by Jones^49^. Concentrations of other mineral elements were measured using inductively coupled plasma optical emission spectroscope (ICP-OES) after nitric acid digestion of plant material. Samples of dried plant material (200 mg, accurately weighed) were digested in a closed vessel microwave oven (ETHOS 1600, Milestone, Italy) using HNO_3_ and H_2_O_2_. Quality control of analytical procedures was performed using the certified reference materials 1515 Apple Leaves and 1568 Rice Flour (National Institute of Standards and Technology, Gaithersburg, USA). Phosphorus and Zn accumulations were calculated by multiplying dry matter (shoots or roots) and their P and Zn concentration, respectively^6^.

### Statistical analysis

Data was subjected to analysis of variance (ANOVA) using the F test (*P* ≤ 0.05) in test version 8.2 (SAS, Cary, NC, USA). The means of treatments for each dependent variable were compared using Tukey’s test (*P* ≤ 0.05). Pearson’s correlation analysis (*P* ≤ 0.05) was performed on the whole dataset to determine which of the dependent variables correlated using the software R (version: 4.0.2, url: https://www.r-project.org/). The “corrplot” package was used to generate the heatmap, using the “cor” and “cor.mtest” functions to generate coefficient matrices and *P*-values, respectively^61^. Sigma Plot 11 software (SYSTAT, San Jose, CA, USA) was used to display data. The present study complies with relevant institutional, national, and international guidelines and legislation.

## Supplementary Information

Supplementary Information.

## Abbreviations

Shoot-DM: Shoot dry matter
Root-DM: Root dry matter
Leaf Area: Total leaf area
P-Leaf: P concentration in diagnostic leaf
Zn-Leaf: Zn concentration in diagnostic leaf
P-Shoot: P content in shoots
Zn-Shoot: Zn content in shoots
P-Root: P content in roots
Zn-Root: Zn content in roots
P-Total Cont: Total P content
Zn-Total Cont: Total Zn content
*A*: Net photosynthesis rate (*A*)
*gs*: Stomatal conductance
*E*: Transpiration
(*k*): Instantaneous carboxylation efficiency
Ci: Sub-stomatal CO_2_ concentration
Fv/Fm: Maximum quantum efficiency of photosystem II
∆F/F m’: Effective quantum efficiency of photosystem II
ETR: Apparent electron transport rate
NQP: Non-photochemical fluorescence quenching
AcidP: Acid phosphatase activity
CarbA: Carbonic anhydrase activity
L-PROT: Leaf protein
L-MDA: Leaf MDA
L-H2O2: Leaf H_2_O_2_
L-CAT: Leaf CAT
L-APX: Leaf APX
L-GPX: Leaf GPX
L-SOD: Leaf SOD
R-PROT: Root protein
R-MDA: Root MDA
R-H2O2: Root H_2_O_2_
R-CAT: Root CAT
R-APX: Root APX
R-GPX: Root GPX
R-SOD: Root SOD
