# Supplementary material for: Unravelling homeostasis effects of phosphorus and zinc nutrition by leaf photochemistry and metabolic adjustment in cotton plants
Source: Sci Rep. 2021 Jul 2;11:13746. doi: 10.1038/s41598-021-93396-1 (PMC8253838; doi:10.1038/s41598-021-93396-1)
Supplement: Supplementary file 1 — Supplementary Information. [file 41598_2021_93396_MOESM1_ESM.docx]

**Supplementary Information:**

**Unravelling homeostasis effects of phosphorus and zinc nutrition by leaf photochemistry and metabolic adjustment in cotton plants**

Elcio Ferreira Santos^a^, Paula Pongrac^b,c^; André Rodrigues Reis^d^, Flávio Henrique Silveira Rabêlo^e^, Ricardo Antunes Azevedo^e^, Philip J. White^f, g, h^; José Lavres^a^

^a^ University of São Paulo, Center for Nuclear Energy in Agriculture, 13416-000, Piracicaba, Brazil

^b^ Biotechnical Faculty, University of Ljubljana, Jamnikarjeva 101, SI-1000 Ljubljana, Slovenia

^c^ Jožef Stefan Institute, Jamova 39, 1000 Ljubljana, Slovenia

^d^ São Paulo State University, 17602-496, Tupã, Brazil

^e^ University of São Paulo, College of Agriculture Luiz de Queiroz, 13418-900, Piracicaba, Brazil

^f^ Ecological Science Group, The James Hutton Institute, Invergowrie, Dundee DD2 5DA, UK

^g^ Distinguished Scientist Fellowship Program, King Saud University, Riyadh 11451, Saudi Arabia

^h^ College of Resources and Environment, Huazhong Agricultural University, Wuhan 430070, China

*Corresponding author.

*E-mail address*: [jlavres@usp.br](mailto:jlavres@usp.br) (J Lavres).

**Supplementary Table 1.** Average concentration (ion homeostasis) of nitrogen (N), potassium (K), calcium (Ca), magnesium (Mg), sulfur (S), boron (B), cupper (Cu), iron (Fe) and manganese (Mn) in the diagnostic leaf (fifth leaf from the top) of cotton (*Gossypium hirsutum*) plants grown in nutrient solution containing different concentrations of phosphorus (P) and zinc (Zn) for 90 days (n = 4).

| Supply | | N | K | Ca | Mg | S | B | Cu | Fe | Mn |
| --- | --- | --- | --- | --- | --- | --- | --- | --- | --- | --- |
| P (mM) | Zn (µM) | ------------------g kg^-1^------------------ | | | | | --------------mg kg^-1^------------ | | | |
| 0.5 | 0.5 | 40.23a | 22.48a | 24.45a | 4.26a | 7.37a | 79.42a | 8.83a | 223.77a | 14.34a |
| 2.0 | 0.5 | 43.22a | 21.96a | 24.77a | 4.51a | 6.26a | 73.87a | 9.10a | 182.04a | 18.12a |
| 4.0 | 0.5 | 40.98a | 20.83a | 26.16a | 4.01a | 8.57a | 71.11a | 7.71a | 121.05a | 12.93a |
| 0.5 | 2.0 | 42.85a | 21.01a | 25.63a | 4.94a | 8.22a | 73.60a | 7.82a | 150.23a | 13.44a |
| 2.0 | 2.0 | 43.15a | 20.83a | 21.17a | 4.06a | 7.02a | 71.30a | 8.16a | 118.35a | 13.71a |
| 4.0 | 2.0 | 43.60a | 21.70a | 25.91a | 4.71a | 7.04a | 57.42a | 7.65a | 133.03a | 19.64a |
| 0.5 | 4.0 | 46.89a | 22.60a | 23.44a | 4.20a | 7.96a | 79.24a | 7.25a | 151.92a | 12.81a |
| 2.0 | 4.0 | 41.88a | 22.48a | 18.59a | 4.00a | 5.78a | 56.10a | 7.76a | 129.49a | 15.36a |
| 4.0 | 4.0 | 41.95a | 21.82a | 19.47a | 4.57a | 5.17a | 63.60a | 6.74a | 139.47a | 17.76a |
| Sufficiency ranges^1^ | | 43.9–46.7 | 19.0–22.6 | 27.5–32.1 | 4.0–4.8 | 8.7–16.5 | 53.6–79.4 | 9.8–22.0 | 80.2–136.5 | 33.4–70.8 |

^1^Sufficiency ranges determined for the nutritional diagnosis of cotton plants based on DRIS norms (NL transformation and F value) by Serra et al (2012). Different letters indicate significant differences for each dependent variable separately (Tukey test at *P* ≤ 0.05; n = 4).

**Supplementary Table 2.** Average concentration (ion homeostasis) of nitrogen (N), phosphorus (P) potassium (K), calcium (Ca), magnesium (Mg), sulfur (S), boron (B), cupper (Cu), iron (Fe), manganese (Mn) and zinc (Zn) in shoots and roots of cotton (*Gossypium hirsutum*) plants grown in nutrient solution containing different concentrations of phosphorus (P) and zinc (Zn) for 90 days (n = 4).

| Supply | | N | P | K | Ca | Mg | S | B | Cu | Fe | Mn | Zn |
| --- | --- | --- | --- | --- | --- | --- | --- | --- | --- | --- | --- | --- |
| P (mM) | Zn (µM) | -----------------------g kg^-1^------------------------ | | | | | | ---------------------mg kg^-1^------------------ | | | | |
|  |  | Shoots | | | | | | | | | | |
| 0.5 | 0.5 | 24.81a | 1.45e | 8.78a | 20.26a | 7.34a | 10.85a | 55.52a | 5.75a | 768.51a | 24.44a | 12.46e |
| 2.0 | 0.5 | 17.74a | 11.84b | 6.37a | 19.36a | 7.27a | 10.23a | 52.97a | 4.83a | 778.21a | 24.36a | 13.24e |
| 4.0 | 0.5 | 18.67a | 27.07a | 6.99a | 21.93a | 7.64a | 10.38a | 59.61a | 6.29a | 661.40a | 24.47a | 13.45e |
| 0.5 | 2.0 | 28.37a | 0.84f | 9.27a | 25.26a | 7.79a | 12.19a | 53.15a | 4.14a | 788.43a | 29.89a | 38.12b |
| 2.0 | 2.0 | 19.45a | 9.70c | 6.63a | 22.74a | 6.99a | 10.32a | 49.10a | 4.02a | 605.92a | 28.20a | 36.34b |
| 4.0 | 2.0 | 12.95a | 11.04b | 6.93a | 23.76a | 4.66a | 10.41a | 40.24a | 4.67a | 663.69a | 28.20a | 20.80c |
| 0.5 | 4.0 | 22.92a | 8.48d | 8.35a | 23.40a | 9.29a | 10.36a | 47.71a | 4.59a | 640.19a | 26.02a | 75.78a |
| 2.0 | 4.0 | 25.75a | 10.84c | 9.96a | 25.32a | 9.01a | 10.67a | 52.06a | 5.25a | 611.43a | 29.60a | 43.88b |
| 4.0 | 4.0 | 14.41a | 13.55b | 6.26a | 21.16a | 6.25a | 10.28a | 47.90a | 5.76a | 625.63a | 26.25a | 38.46c |
|  |  | Roots | | | | | | | | | | |
| 0.5 | 0.5 | 19.89a | 2.71c | 4.54a | 8.31a | 2.25a | 5.61a | 30.68a | 0.70a | 1740.84a | 9.27a | 7.62d |
| 2.0 | 0.5 | 19.63a | 5.59b | 4.25a | 8.83a | 2.52a | 5.13a | 27.14a | 0.30a | 1314.48a | 8.88a | 7.65d |
| 4.0 | 0.5 | 20.34a | 5.74b | 4.07a | 8.55a | 2.38a | 6.04a | 30.24a | 0.98a | 1788.06a | 8.58a | 8.35d |
| 0.5 | 2.0 | 19.99a | 1.41c | 4.26a | 7.91a | 1.64a | 4.51a | 20.76a | 0.30a | 1517.45a | 8.43a | 13.99c |
| 2.0 | 2.0 | 18.50a | 5.84b | 4.28a | 8.05a | 1.46a | 4.85a | 21.72a | 0.60a | 1707.91a | 7.94a | 19.25c |
| 4.0 | 2.0 | 16.32a | 6.71b | 4.82a | 8.61a | 2.01a | 4.66a | 32.81a | 0.31a | 1521.19a | 8.91a | 23.08b |
| 0.5 | 4.0 | 16.51a | 2.85c | 4.18a | 8.73a | 2.17a | 4.01a | 24.57a | 0.21a | 1545.73a | 7.00a | 26.38b |
| 2.0 | 4.0 | 16.09a | 7.10b | 4.33a | 8.56a | 1.95a | 5.04a | 28.95a | 0.38a | 1598.01a | 7.79a | 29.42b |
| 4.0 | 4.0 | 18.18a | 15.22a | 4.20a | 7.88a | 1.61a | 4.26a | 26.22a | 0.39a | 1740.69a | 7.35a | 40.12a |

Different letters indicate significant differences for each dependent variable separately (Tukey test at *P* ≤ 0.05; n = 4).
